# Transcriptional Profile and Structural Conservation of SUMO-Specific Proteases in *Schistosoma mansoni*

**DOI:** 10.1155/2012/480824

**Published:** 2012-10-18

**Authors:** Roberta Verciano Pereira, Fernanda Janku Cabral, Matheus de Souza Gomes, Liana Konovaloff Jannotti-Passos, William Castro-Borges, Renata Guerra-Sá

**Affiliations:** ^1^Departamento de Ciências Biológicas/Núcleo de Pesquisas em Ciências Biológicas, Instituto de Ciências Exatas e Biológicas, Universidade Federal de Ouro Preto, Campus Morro do Cruzeiro, 35400-000 Ouro Preto, MG, Brazil; ^2^Departamento de Parasitologia, Instituto de Ciências Biomédicas, Universidade de São Paulo, Av. Lineu Prestes, 1374-Butantan, 05508-900 São Paulo, SP, Brazil; ^3^Instituto de Genética e Bioquímica, Universidade Federal de Uberlândia, Av. Getúlio Vargas, Palácio dos Cristais-Centro, 38700-126 Patos de Minas, MG, Brazil; ^4^Fundação Oswaldo Cruz, Centro de Pesquisas René Rachou, Laboratório de Malacologia, Av. Augusto de Lima, 1715-Barro Preto, 30190-002 Belo Horizonte, MG, Brazil

## Abstract

Small ubiquitin-related modifier (SUMO) is involved in numerous cellular processes including protein localization, transcription, and cell cycle control. SUMOylation is a dynamic process, catalyzed by three SUMO-specific enzymes and reversed by Sentrin/SUMO-specific proteases (SENPs). Here we report the characterization of these proteases in *Schistosoma mansoni*. Using *in silico* analysis, we identified two SENPs sequences, orthologs of mammalian SENP1 and SENP7, confirming their identities and conservation through phylogenetic analysis. In addition, the transcript levels of *Smsenp1/7* in cercariae, adult worms, and *in vitro* cultivated schistosomula were measured by qRT-PCR. Our data revealed upregulation of the *Smsenp1/7* transcripts in cercariae and early schistosomula, followed by a marked differential gene expression in the other analyzed stages. However, no significant difference in expression profile between the paralogs was observed for the analyzed stages. Furthermore, in order to detect deSUMOylating capabilities in crude parasite extracts, *Sm*SENP1 enzymatic activity was evaluated using SUMO-1-AMC substrate. The endopeptidase activity related to SUMO-1 precursor processing did not differ significantly between cercariae and adult worms. Taken together, these results support the developmentally regulated expression of SUMO-specific proteases in *S. mansoni*.

## 1. Introduction

Reversible posttranslational modification by ubiquitin and ubiquitin-like proteins (Ubls) plays a crucial role in dynamic regulation of protein function, fate, binding partners, activity, and localization. Small ubiquitin-related modifier (SUMO) is a member of Ubls family that is covalently attached to lysine residues of their protein targets in cells, altering function and subcellular localization [1]. SUMOylation has been implicated in the regulation of numerous biological processes including transcription [2] and cell cycle control [3]. SUMO conjugation mechanism is a highly dynamic and reversible process closely related to ubiquitylation. The sequential actions of E1, E2, and E3 enzymes catalyze the attachment of SUMO to target proteins, while deconjugation is promoted by SUMO-specific proteases. In addition, like other Ubls, SUMO polypeptide is synthesized as an inactive precursor, which requires a C-terminal cleavage to expose the glycine residue for substrate conjugation. This process is also catalyzed by SUMO-specific protease through its hydrolase activity. The catalytic activity is maintained within a highly conserved 200 amino acid region at the C-terminus of the proteases [4–6].

Recently, the importance of reversible SUMOylation for normal cell physiology has been demonstrated. Excessive SUMO conjugation using knockouts for either SENP1 or SENP2 induced an embryonic lethal phenotype [7, 8] and deregulation of either SUMO conjugation or deconjugation can contribute to cancer progression as reviewed by [9]. In humans there are six SENP enzymes (SENP1, -2, -3, -5, -6, and -7) that deconjugate mono-SUMOylated proteins or disassemble polymeric SUMO side chains, while in *Saccharomyces cerevisiae* there are only two deSUMOylating enzymes, Ulp1 and Ulp2. Based on these activities and affinities for SUMO isoforms, SENPs can be categorized into three independent subfamilies: SENP1 and SENP2; SENP3 and SENP5; SENP6 and SENP7 [9].

The initial molecular characterization of SUMOylation pathway in *S. mansoni* showed the presence of two SUMO paralogs called SMT3C and SMT3B [10], suggesting a variety of targeted substrates for these modifications. Recently, our group reported the conservation of SUMO conjugating enzyme *Sm*UBC9 by phylogeny, primary and modeled tertiary structures, as well as mRNA transcription and protein levels throughout the *S. mansoni* life cycle. Our data showed that *Smubc9* transcription levels are upregulated in early schistosomula, suggesting the utilization of this posttranslational modification mechanism *S. mansoni* development, particularly at the initial phase of invasion of the vertebrate host [11].

To extend the investigation of the SUMOylation pathway in *S. mansoni*, we firstly retrieved through homology-based searches putative *Sm*SENPs using the publically available *S*. *mansoni* databases. Furthermore, the levels of *Smsenp1/7* transcripts were evaluated by qRT-PCR using mRNA from cercariae, adult worms and mechanically-transformed schistosomula (MTS) *in vitro*-cultivated during 3.5 h, 1, 2, 3, 5, and 7 days. We also measured the endopeptidase activities of *Sm*SENPs in cercariae and adult worm crude extracts. The present study reveals differences in the gene expression profile of *Smsenp1/7* during schistosomula development. In addition, similar *SmSENP1* activity was observed in cercariae and adult worms, suggesting the importance of this proteolytic activity during the parasite's life cycle.

## 2. Materials and Methods

### 2.1. Ethics Statement

All experiments involving animals were authorized by the Ethical Committee for Animal Care of Federal University of Ouro Preto, and were in accordance with the national and international regulation accepted for laboratory animal use and care.

### 2.2. Parasites. *S. mansoni*

LE strain was maintained by routine passage through *Biomphalaria glabrata* snails and BALB/c mice. The infected snails were induced to shed cercariae under light exposure for 2 h and the cercariae were recovered by sedimentation on ice. Adult worm parasites were obtained by liver perfusion of mice after 50 days of infection. Mechanically transformed schistosomula (MTS) were prepared as described by Harrop and Wilson [12]. Briefly, cercariae were recovered, and washed in RPMI 1640 medium (Invitrogen), before vortexing at maximum speed for 90 s and immediately cultured during 3.5 h at 37°C, 5% CO_2_. Then the recovered schistosomula were washed with RPMI 1640 until no tails were detected. For subsequent incubations, the parasites were maintained in M169 medium supplemented with 10% FBS, penicillin, and streptomycin at 100 *μ*g/mL and 5% Schneider's medium [13] at 37°C on 5% CO_2_ during 3.5 h, 1, 2, 3, 5, and 7 days.

### 2.3. Identification and Computational Analysis of *Sm*SENP1/7

*Sm*SENP1/7 sequences were retrieved from the *S. mansoni* genome database version 5.0 available at http://www.genedb.org/genedb/smansoni/ through BLAST searches. Amino acid sequences from *Drosophila melanogaster*, *Caenorhabditis elegans* and *Homo sapiens SENP* orthologs were used as queries. The BLASTp algorithm, underpinned by the Pfam (v26.0), allowed detection of conserved protein domains or motifs from *S. mansoni* sequences. Multiple alignments of *Sm*SENP1/7 were performed by ClustalX 2.0 and phylogenetic analysis was conducted in MEGA 5 [14]. A phylogenetic tree of these sequences was inferred using the Neighbor-Joining method [15]. The bootstrap consensus tree inferred from 1000 replicates was used to represent the evolutionary history of the taxa analyzed. Branches corresponding to partitions reproduced in less than 50% bootstrap replicates are collapsed. The percentage of replicate trees in which the associated taxa clustered together in the bootstrap test (1000 replicates) is shown next to the branches. The tree was drawn to scale, with branch lengths in the same units as those of the evolutionary distances used to infer the phylogenetic tree. All positions containing gaps and missing data were eliminated from the dataset.

### 2.4. RNA Preparation and Expression Analysis of *Sm*senp1/7 by qRT-PCR

Total RNA from adult worms, cercariae, and schistosomula was obtained using a combination of the Trizol reagent (GIBCO-BRL) and chloroform for extraction, and then on column purified using the “SV total RNA Isolation System” (Promega, Belo Horizonte, Brazil). The preparation was treated with RNase-free DNase I in 3 different rounds by decreasing enzyme concentration (RQ1 DNase; Promega). RNA was quantified using a spectrophotometer and an aliquot containing 1 *μ*g of total RNA reverse transcribed using an oligodT primer from the Thermoscript RT-PCR System (Invitrogen São Paulo, Brazil) as described by the manufacturer. The efficiency of DNAse I treatment was evaluated by PCR amplification of the cDNA reaction mix without the addition of the Thermoscript enzyme. *S. mansoni* specific SENP1/7 primers were designed using the program GeneRunner for *Sm*SENP1 (GeneDB access number Smp_033260.2) (forward 5′-AGGAAACGGAGGCGGGATTC-3′, reverse 5′-ACACTGGAGACACGGGATGAGC-3′) and for *Sm*SENP7 (GeneDB access number Smp_159120) (forward 5′-TCAGTTACACGGCCCTTTATC-3′, reverse 5′-CCTGAGAAGTGGATGCGATC-3′). Reverse-transcribed cDNA samples were used as templates for PCR amplification using SYBR Green Master Mix UDG-ROX (Invitrogen) and 7300 Real Time PCR System (Applied Biosystems, Rio de Janeiro, Brazil). Specific primers for *S. mansoniα*-tubulin were used as an endogenous control (GenBank access number M80214) (forward 5′-CGTATTCGCAAGTTGGCTGACCA-3′, reverse 5′-CCATCGAAGCGCAGTGATGCA-3′) [16]. The efficiency of each pair of primers was evaluated according to the protocol developed by the Applied Biosystems application (cDNA dilutions were 1 : 10, 1 : 100 and 1 : 1000). For all investigated transcripts three biological replicates were performed and their gene expression normalized against the *α*-tubulin transcript according to the 2^−Δ*C*_*t*_^ method [17] using the Applied Biosystems 7300 software.

### 2.5. Endopeptidase Activity

To determine the enzymatic activity of SENP proteases present in adult worms and cercariae crude extracts we used the fluorogenic substrate SUMO-1-AMC that is specific for SENP1 (Enzo Life Sciences, São Paulo, Brazil). In these assays 10 *μ*g of total protein were used and 0,45 *μ*M of the fluorogenic substrate in 50 mM Tris-HCl pH 8, 10 mM MgCl_2_ ± 0,45 *μ*M SUMO-1-aldehyde (Enzo Life Sciences) to control for specific enzyme inhibition. Each enzymatic assay was conducted in a final volume of 100 *μ*L for both extracts, followed by 60 minutes incubation at 37°C. The reaction was stopped by addition of 2 mL of 99.5% ethanol. The fluorometric readings were taken at wavelengths of 380 nm (excitation) and 440 nm (emission) in a spectrofluorimeter (Turner QuantechTM Fluorometer), and the results expressed in fluorescence units per *μ*g of total protein.

### 2.6. Statistical Analysis

Statistical analysis was performed using GraphPad Prism version 5.0 software package (Irvine, CA, USA). Normality of the data was established using one way analysis of variance (ANOVA). Tukey post tests were used to investigate significant differential expression of SUMO proteases throughout the investigated stages. In all cases, the differences were considered significant when *P* values were <0.05.

## 3. Results

### 3.1. SUMO Specific-Proteases: The Conservation of *Sm*SENP1/7

**Figure 1:**
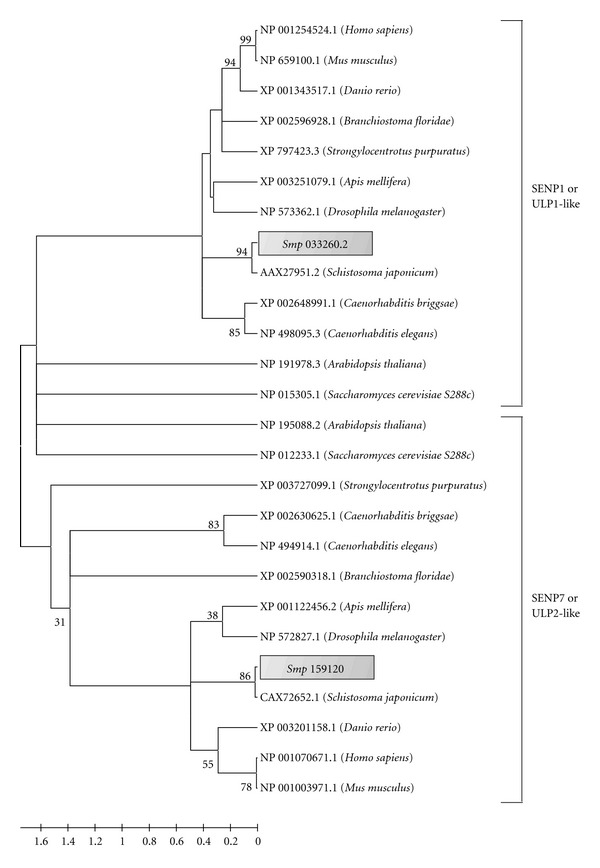
Consensus phylogenetic tree based on amino acid sequences of *Sm*SENPs. The tree construction and analysis of bootstrap were performed using ClustalX 2.0 and MEGA 4.0. For the consensus tree and reliability of the branches formed test was used phylogenetic bootstrap using 1000 replicates for each sequence, and 50% the minimum for considering the branch reliably. Organisms: Hs (*Homo sapiens*), Mm (*Mus musculus*), Rn (*Rattus norvegicus*), Xl (*Xenopus laevis*), Sm (*Schistosoma mansoni*), Sj (*Schistosoma japonicum*), Dm (*Drosophila melanogaster*), Cb (*Caenorhabditis briggsae*) and Ce (*Caenorhabditis elegans*).

*In silico* analysis of *Sm*SENPs revealed the conserved putative domains of SUMO proteases in *S. mansoni*. The parasite database has two sequences encoding putative SENPs: Smp_033260.2 (putative SENP1) and Smp_159120 (putative SENP7). SENP1 has an alternative spliced form annotated as Smp_033260.1. We evaluated the conservation of these proteins throughout evolution using a phylogenetic approach. Our results showed that each SENP was grouped in 2 distinct clades, reinforcing their structural conservation among the thirteen analyzed orthologs (Figure 1).

**Figure 2:**
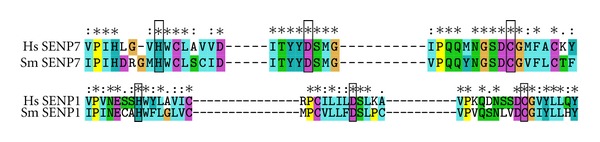
*S. mansoni* has two predicted SUMO-Specific Proteases. ClustalX 2.0 alignment of SENP1 and SENP7 catalytic residues from human and *S. mansoni*. The domains position of proteins in *S. mansoni* were based on Pfam database as well as the *e*-value score. The grey boxes represent the conserved domains based on Pfam database. Aligned catalytic residues are denoted by the black box. ∗,  :,  and  . indicate, respectively, identical residues, highly conserved amino acid substitution, and conserved amino acid substitution.

To confirm that these predicted proteases are cysteine-family members, analyses using the Pfam protein domain database were conducted. For both *Sm*SENPs entries a conserved Peptidase_C48 domain was revealed. Our *in silico* analysis demonstrated *Sm*SENP1 more closely related to *Hs*SENP1 (GenBank access number NP_055369.1), whereas *Sm*SENP7 is related to *Hs*SENP7 (GenBank access number NP_065705.3). Alignment of their catalytic domains with the human SENPs showed the presence of the three essential catalytic residues (Cys-His-Asp), highlighted in Figure 2.

### 3.2. *Sm*senp1/7 Are Differentially Expressed in *S. mansoni*

**Figure 3:**
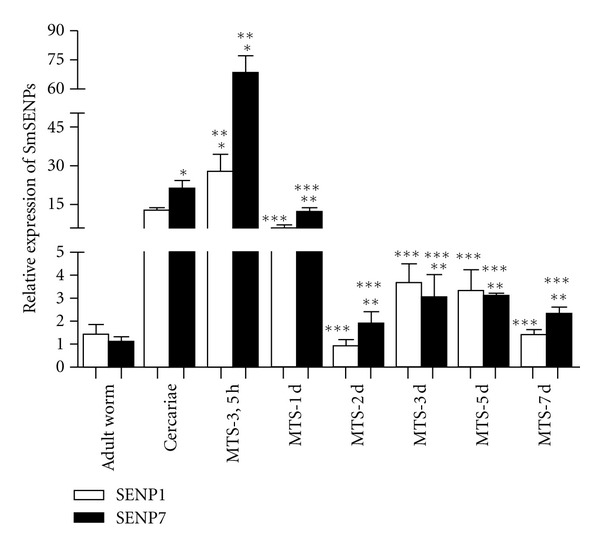
*Sm*SENPs are differentially expressed throughout the *S. mansoni* life cycle. The mRNA expression levels were measured based on three replicates, for each of the following stages: adult worms, cercariae, MTS-3.5 h, 1, 2, 3, 5, and 7 days using quantitative RT-PCR. Expression levels were calibrated according to the comparative 2^−Δ*C*_*t*_^ method, using the constitutively expressed *Sm*α**-tubulin as an endogenous control (one-way variance analysis followed by Tukey pairwise comparison *P* < 0.05). *Different from adult worm, **different from cercariae, and ***different from MTS-3.5 h.

The gene expression profile of *Smsenp1* and *Smsenp7* was determined using qRT-PCR. We designed specific primers to amplify *Smsenp1* and *Smsenp7* transcripts in the cercariae-schistosomula transition and adult worm (Figure 3). We observed that *Smsenp1* and *Smsenp7* transcripts are expressed in basal levels from MTS-2d to 7 days and adult worms. The levels of the SENPs transcripts were significantly higher in MTS-3.5 h being approximately 10-fold higher when compared to levels in adult worms and during schistosomula development (MTS-2, 3, 5, and 7 days) and 3-fold higher when compared to cercariae and MTS-1d. We also observed that *Smsenp1* and *Smsenp7* transcription levels were not significantly different (*P* < 0.05) within a given stage, except for MTS-3.5 h where *Smsenp7* levels are 3 fold-higher than *Smsenp1*.

### 3.3. *Sm*SENP1 Enzymatic Activity in Cercariae and Adult Worms

**Figure 4:**
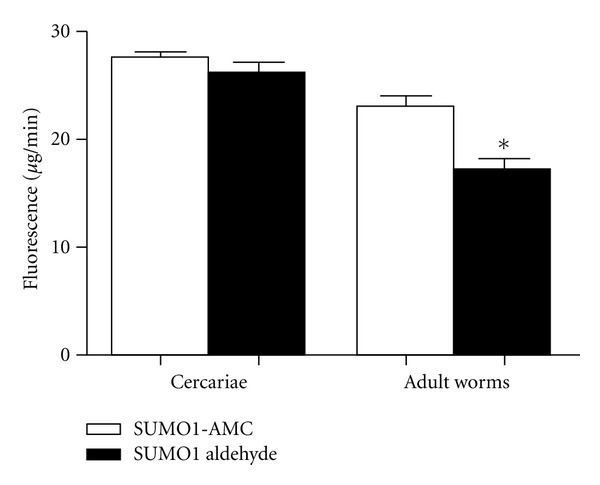
*Sm*SENP1 enzymatic activity in *S. mansoni*. The fluorescence levels were measured based on three replicates, in cercariae and adult worm stages. Statistical analysis was performed using *t* tests followed by unpaired test *P* < 0.05. *Different from cercariae.

*In vitro* endopeptidase assays were performed using crude extract from cercariae and adult worms (Figure 4). The *Sm*SENP1 enzymatic activity was slightly higher in cercariae compared to adult worms, although the levels were not significantly different (*P* < 0.05). Enzyme activities were also recorded in the presence of a commercially available *Hs*SENP1 inhibitor but significant differences were not observed in the analyzed stages.

## 4. Discussion

Previous reports from our group showed the presence of two SUMO paralogs (SMT3C and SMT3B) in *S. mansoni*, exhibiting differential expression between larvae and adult worms. The electrophoretic pattern of SUMO-conjugated molecules also differed comparing the analyzed stages [10]. Furthermore, our group demonstrated a differential expression profile for UBC9 throughout the parasite life cycle [11]. The structural SUMO conservation in *S. mansoni* reinforces SUMO as a candidate for a transcription regulator and a degradation signal by facilitating ubiquitylation of certain target proteins during parasite development.

In order to understand the role of SUMOylation in *S. mansoni*, we evaluated the conservation of SUMO proteases and their gene expression profile in cercariae, schistosomula, and adult worm. Firstly, we retrieved, through homology-based searches, two sequences containing conserved domains of SUMO proteases. A phylogenetic approach then revealed two putative proteases closely associated to human SENP1 and SENP7. Alignment of parasite sequences with their human counterparts also demonstrated conservation of the catalytic triad for both proteases [18]. The active site residues within the core domain of Ulp1 and Ulp2 have been proven to be necessary for catalytic activity and *in vivo* function in yeast [19, 20]. Furthermore, Ulp1 lacks obvious sequence similarity to any known deubiquitinating enzyme so it is unlikely that it is able to process ubiquitin-linked substrates [21].

Considering that the SUMOylation pathway dictates the function of a variety of substrates [22], and the existence of two SUMO paralogs in *S. mansoni* [10], it is plausible that the worm utilizes distinct SUMO proteases for SUMO maturation and deSUMOylation of target substrates. Discrimination between SUMO-1 and SUMO-2/3 paralogs may also account for distinct functions of SENP proteases in *S. mansoni*. In mammalian cells, proteomic studies have identified proteins that are selectively modified by either SUMO-1 or SUMO-2/3 [23]. It is well established that SENP1 processes SUMO-1 in preference to SUMO-2, and has a very low catalytic constant for SUMO-3 [24]. In contrast, the recent characterization of the catalytic domain of SENP7 in humans demonstrated a greater deconjugating activity for SUMO-2/3 chains [25]. As discussed by Shen [26] SENP7 acts as a SUMO-2/3 specific protease that is likely to regulate the metabolism of polySUMO-2/3 rather than SUMO-1 conjugation *in vivo*.

In the present investigation, the transcriptional profile of SUMO proteases was also evaluated by qPCR, using total RNA from distinct stages of the *S. mansoni* life cycle. The gene expression data revealed similar expression profile for *Smsenp1* and *Smsenp7* at any given stage, but with a remarkable differential expression for both proteases comparing the larvae and adult stages. The differential gene expression profile observed for *Smsenp1/7* throughout the *S. mansoni* life cycle attests for the distinct patterns of SUMO conjugates observed during parasite development [10]. PolySUMOylation has been reported to increase during heat-shock treatment and stress conditions, with SENP7 being responsible for dismantling SUMO polymers [25, 27]. Given that the levels of *Smsenp1/7* transcripts were significantly higher in cercariae and MTS-3.5 h, we suggest a stress-induced expression of the SUMOylation machinery during the parasite's body adaptation to the new metabolic and morphological alterations it undergoes [28]. Our findings are also in agreement with Pereira [11] which demonstrated a similar pattern of gene expression profile for *Smubc9* in the aforementioned parasite stages.

We have not observed a positive correlation between differential transcriptional levels of *Smsenp1* and enzymatic activity evaluated using synthetic SUMO-1-AMC substrate. The levels of enzymatic activity for *SmSENP1* were slightly higher in extracts of cercariae compared to adult worms. In the presence of a specific inhibitor, no significant change in enzymatic activity was detected. This may account for the limited amino acid sequence identity (28%) shared between *SmSENP1* and its human ortholog, from which the synthesis of SUMO-1 aldehyde is based. Additional studies aimed at elucidating substrate selection of *SmSENP1/7* in *S. mansoni* and a detailed kinetic analysis of their enzymatic activities, particularly during the cercarie to schistosomula transition may provide further insights into the biological activities of these enzymes and the role of SUMO conjugation in *S. mansoni*.

## References

[1] Gareau JR, Lima CD (2010). The SUMO pathway: emerging mechanisms that shape specificity, conjugation and recognition. *Nature Reviews Molecular Cell Biology*.

[2] Desterro JMP, Rodriguez MS, Hay RT (1998). SUMO-1 modification of I*κ*B*α* inhibits NF-*κ*B activation. *Molecular Cell*.

[3] Ulrich HD (2009). Regulating post-translational modifications of the eukaryotic replication clamp PCNA. *DNA Repair*.

[4] Mukhopadhyay D, Dasso M (2007). Modification in reverse: the SUMO proteases. *Trends in Biochemical Sciences*.

[5] Kim JH, Baek SH (2009). Emerging roles of desumoylating enzymes. *Biochimica et Biophysica Acta*.

[6] Xu Z, Chan HY, Lam WL (2009). SUMO proteases: redox regulation and biological consequences. *Antioxidants and Redox Signaling*.

[7] Cheng J, Kang X, Zhang S, Yeh ETH (2007). SUMO-specific protease 1 is essential for stabilization of HIF1*α* during hypoxia. *Cell*.

[8] Chiu SY, Asai N, Costantini F, Hsu W (2008). SUMO-specific protease 2 is essential for modulating p53-Mdm2 in development of trophoblast stem cell niches and lineages. *PLOS Biology*.

[9] Bawa-Khalfe T, Yeh ETH (2010). SUMO losing balance: SUMO proteases disrupt SUMO homeostasis to facilitate cancer development and progression. *Genes and Cancer*.

[10] Cabral FJ, Pereira OS, Silva CS, Guerra-Sá R, Rodrigues V (2008). *Schistosoma mansoni* encodes SMT3B and SMT3C molecules responsible for post-translational modification of cellular proteins. *Parasitology International*.

[11] Pereira RV, Cabral FJ, Gomes MS (2011). Molecular characterization of SUMO E2 conjugation enzyme: differential expression profile in *Schistosoma mansoni*. *Parasitology Research*.

[12] Harrop R, Wilson RA (1993). Protein synthesis and release by cultured schistosomula of *Schistosoma mansoni*. *Parasitology*.

[13] Basch PF, DiConza JJ (1977). In vitro development of *Schistosoma mansoni* cercariae. *Journal of Parasitology*.

[14] Kumar S, Tamura K, Nei M (2004). MEGA3: integrated software for Molecular Evolutionary Genetics Analysis and sequence alignment. *Briefings in bioinformatics*.

[15] Saitou N, Nei M (1987). The neighbor-joining method: a new method for reconstructing phylogenetic trees. *Molecular Biology and Evolution*.

[16] Webster PJ, Seta KA, Chung SC, Mansour TE (1992). A cDNA encoding an *α*-tubulin from *Schistosoma mansoni*. *Molecular and Biochemical Parasitology*.

[17] Livak KJ, Schmittgen TD (2001). Analysis of relative gene expression data using real-time quantitative PCR and the 2-ΔΔCT method. *Methods*.

[18] Li SJ, Hochstrasser M (2003). The Ulp1 SUMO isopeptidase: distinct domains required for viability, nuclear envelope localization, and substrate specificity. *Journal of Cell Biology*.

[19] Li SJ, Hochstrasser M (1999). A new protease required for cell-cycle progression in yeast. *Nature*.

[20] Strunnikov AV, Aravind L, Koonin EV (2001). Saccharomyces cerevisiae SMT4 encodes an evolutionarily conserved protease with a role in chromosome condensation regulation. *Genetics*.

[21] Li SJ, Hochstrasser M (2000). The yeast ULP2 (SMT4) gene encodes a novel protease specific for the ubiquitin-like Smt3 protein. *Molecular and Cellular Biology*.

[22] Rosas-Acosta G, Russell WK, Deyrieux A, Russell DH, Wilson VG (2005). A universal stategy for proteomic studies of SUMO and other ubiquitin-like modifiers. *Molecular and Cellular Proteomics*.

[23] Vertegaal ACO, Andersen JS, Ogg SC, Hay RT, Mann M, Lamond AI (2006). Distinct and overlapping sets of SUMO-1 and SUMO-2 target proteins revealed by quantitative proteomics. *Molecular and Cellular Proteomics*.

[24] Shen LN, Dong C, Liu H, Naismith JH, Hay RT (2006). The structure of SENP1-SUMO-2 complex suggests a structural basis for discrimination between SUMO paralogues during processing. *Biochemical Journal*.

[25] Lima CD, Reverter D (2008). Structure of the human SENP7 catalytic domain and poly-SUMO deconjugation activities for SENP6 and SENP7. *Journal of Biological Chemistry*.

[26] Shen LN, Geoffroy MC, Jaffray EG, Hay RT (2009). Characterization of SENP7, a SUMO-2/3-specific isopeptidase. *Biochemical Journal*.

[27] Golebiowski F, Matic I, Tatham MH (2009). System-wide changes to sumo modifications in response to heat shock. *Science Signaling*.

[28] Lawson JR, Wilson RA (1980). Metabolic changes associated with the migration of the schistosomulum of *Schistosoma mansoni* in the mammal host. *Parasitology*.

